# Orbitale Schussverletzungen

**DOI:** 10.1007/s00347-020-01195-2

**Published:** 2020-08-26

**Authors:** C. S. Mayer, S. Bohnacker, J. Storr, M. Klopfer, S. A. Cordeiro, R. Khoramnia

**Affiliations:** 1 Univ.-Augenklinik Heidelberg, Medizinische Fakultät Heidelberg der Universität Heidelberg, Im Neuenheimer Feld 400, 69120 Heidelberg, Deutschland; 2 Diakonissenkrankenhaus, Diakonissenstr. 28, 76199 Karlsruhe, Deutschland; 3 Universitätsaugenklinik, TU München, Ismaningerstr. 22, 81675 München, Deutschland; 4 Klinik für allgemeine Psychiatrie, Universitätsklinikum Heidelberg, Voßstr. 4, 69115 Heidelberg, Deutschland

**Keywords:** Waffe, Projektil, Suizid, Trauma, Bulbusruptur, Weapon, Projectile, Suicide, Trauma, Eyeball rupture

## Abstract

**Hintergrund:**

Schussverletzungen kommen im Bereich der Ophthalmologie glücklicherweise selten vor. Die Behandlung eines betroffenen Patienten ist sowohl ophthalmologisch als auch emotional eine besondere Herausforderung.

**Methodik:**

Wir berichten über 7 konsekutive Fälle von Patienten mit einer orbitalen Schussverletzung, die über einen Zeitraum von 11 Jahren in einem Universitätsklinikum vorstellig wurden. Ausgewertet wurden der Schadenshergang mit beteiligter Waffe, die Schussart, das Verletzungsmuster, die kraniale Bildgebung, die Therapie und der Verlauf.

**Ergebnisse:**

Insgesamt konnten 7 Fälle von Schussverletzungen durch Waffen im Zeitraum von 2007 bis 2018 dokumentiert und ausgewertet werden. Alle 7 Patienten waren männlich. Das Alter betrug im Mittel 44 ± 27,5 Jahre. Fünfmal kam es durch einen Suizidversuch zu den Verletzungen, zweimal durch einen Unfall. Bis auf eine Schussverletzung mit einer Armbrust wurden Feuerwaffen verwendet. Viermal kam es dabei zu einem Steckschuss, zweimal zu einem Durchschuss und einmal zu einem Prellschuss. Der Endvisus lag bei den betroffenen Augen einmal bei einer einseitigen und ein weiteres Mal bei einer beidseitigen Amaurose, bei einem Patienten bei 1/35 MTV und bei 4 Patienten zwischen 0,2 und 0,7. Kein Patient ist an den unmittelbaren Folgen der Schussverletzung gestorben.

**Diskussion:**

Schussverletzungen sind in Deutschland selten und haben meist einen suizidalen Hintergrund. Das Verletzungsmuster im Bereich der okulären Strukturen ist sehr variabel. Ein Rekonstruktionsversuch lohnt sich prinzipiell immer. Bei der Versorgung der Patienten ist eine interdisziplinäre Zusammenarbeit von Ophthalmologen und Neuro- und MKG- bzw. HNO-Chirurgen sowie Psychiatern notwendig.

## Abstract (en)

**Background:**

Injuries caused by firearms are fortunately rare in the field of ophthalmology. The treatment of an affected patient is a special challenge both ophthalmologically and emotionally.

**Methods:**

We report on seven consecutive cases of patients with an orbital gunshot injury who presented in a university hospital over a period of 11 years. The course of events leading to the injury with the weapon involved, the type of projectile, the injury pattern, cranial imaging, treatment and course were evaluated.

**Results:**

A total of seven cases of injuries caused by firearms could be documented and evaluated in the period 2007–2018. All seven patients were male. The average age was 44 ± 27.5 years. Of the injuries five were caused by a suicide attempt and two by an accident. Firearms were used except for one injury caused by a crossbow. There was a retained projectile in four of the cases, the bullet went through the body in two cases and one of the cases presented with a ricochet shot. The final visual acuity was unilateral amaurosis in 1 case and bilateral amaurosis in another case, 1/35 (measured at 1 m) in 1 case, while it varied between 0.2 and 0.7 in the other 4 cases. No patient died as a direct consequence of the firearm injury.

**Conclusion:**

Injuries caused by firearms are relatively rare in Germany and mostly have a suicidal background. The pattern of the injury of the ocular structures is very variable. A reconstruction attempt is principally recommended. In the care of patients interdisciplinary cooperation between ophthalmologists and neurosurgeons, orofacial surgeons, ear nose and throat (ENT) surgeons and psychiatrists is necessary.

Schussverletzungen der Orbitae, Augen und der Augenanhangsgebilde kommen in Deutschland selten vor. Dies spiegelt sich auch in der geringen Anzahl deutschsprachiger Publikationen zu diesem Thema wider. Die meisten publizierten Berichte sind Einzelfallbeschreibungen [8, 18].

## Hintergrund und Fragestellung

Der Zugriff auf Schusswaffen ist in Deutschland stark beschränkt. Dagegen wird der Zugriff in anderen Ländern freier gehandhabt. So ist in den USA beispielsweise durch den 2. Zusatzartikel zur Verfassung vom 15.12.1791 das Recht, Waffen zu besitzen, sogar verfassungsrechtlich geschützt. In den meisten Bundesstaaten sind Waffen daher frei verkäuflich. Aber auch international wird zu dem Thema vergleichsweise wenig publiziert [1, 4, 7, 20]. In einem Großteil der europäischen Länder sind der Erwerb, der Besitz und der Schusswaffengebrauch durch entsprechende Gesetze reglementiert. Schusswaffen können in Deutschland beispielsweise laut §§ 4–9 Waffengesetz nur unter bestimmten Voraussetzungen erworben werden. Verstöße gegen das Gesetz sind meist ein Vergehen, also eine Straftat nach § 52 der Strafvorschriften des WaffG. Die Restriktionen für den freien Zugriff auf Schusswaffen einerseits und die Tatsache andererseits, dass okuläre Schussverletzungen oft mit Begleitverletzungen einhergehen, die einen letalen Ausgang nach sich ziehen, machen orbitale Schussverletzungen zu einer seltenen augenärztlichen Diagnose [19]. Ein sehr heterogenes Verletzungsmuster und eine komplexe Gesamtsituation nach diesem Ereignis erschweren zusätzlich wissenschaftliche Auswertungen. An der Versorgung betroffener Patienten sind oftmals unterschiedliche medizinische Fachdisziplinen beteiligt. Diese bedarf in der Regel einer interdisziplinären Zusammenarbeit von Ophthalmologen mit Kollegen der Hals-Nasen-Ohrenheilkunde (HNO), Mund-Kiefer-Gesichtschirurgie (MKG), (Neuro‑)Chirurgie und Psychiatrie. Gerade weil okuläre Schussverletzungen in unserem Umfeld selten vorkommen, stellt die Behandlung eines betroffenen Patienten sowohl ophthalmologisch als auch emotional eine besondere Herausforderung dar.

Im Folgenden berichten wir über 7 konsekutive Fälle, die in einem Zeitraum von 11 Jahren vorstellig wurden.

## Methodik

An einem Zentrum der Maximalversorgung wurden retrospektiv aus dem Krankenregister alle konsekutiven Patienten im Zeitraum von 2007 bis 2018 ermittelt, die eine Schussverletzung durch eine Waffe mit Augen- und/oder Orbitabeteiligung erlitten und diese mindestens bis zur Vorstellung beim Augenarzt überlebten. Als Definition einer Schussverletzung galt hier „eine Verletzung, die durch ein Geschoss (Pfeil oder Projektil) verursacht wird“.

Im waffenrechtlichen Sinn ist die Armbrust, im Gegensatz zum Bogen, Schusswaffen gleichgestellt. Damit finden die für Schusswaffen geltenden Regelungen auch auf die Armbrust Anwendung. Das Schießen mit einer Armbrust ist gem. § 1 Abs. 3 und § 2 Abs. 1 WaffG als Umgang mit einer Waffe einzuordnen. Jedoch gehört sie zu den freien Waffen: Erwerb, Besitz, Handel und Herstellung sind somit erlaubt.

Nicht berücksichtigt wurden in dieser Arbeit milde Kontusionen sowie Perforationen oder Projektilschäden, die nicht durch eine Waffe verursacht worden sind, und Schussverletzungen, die keine Augenbeteiligung zur Folge hatten. Es wurden der Schadenshergang mit beteiligter Waffe, das Verletzungsmuster, die kraniale Bildgebung, die Anamnese, die Therapie und der Verlauf (inklusive das Überleben) ausgewertet. Darüber hinaus wurde die Art des Schusses nach Weg und Verbleib des Projektils eingeteilt:

- Durchschuss (Projektil durchdringt den Körper in der Regel mit einer Ein- sowie einer Austrittspforte),
- Steckschuss (das Projektil verbleibt im Körper),
- Streifschuss (verläuft tangential zur Körperoberfläche, sodass das Geschoss eine grabenförmige Wunde aufreißt, ohne jedoch in den Körper zu einzudringen),
- Prellschuss (ohne äußere Hautwunde, Geschoss dringt nicht ein).

Die Untersuchungen und Auswertungen wurden im Einklang mit nationalem Recht sowie in Übereinstimmung mit der Deklaration von Helsinki von 1975 (in ihrer aktuellen, überarbeiteten Fassung) durchgeführt.

## Ergebnisse

Insgesamt konnten 7 Fälle von Schussverletzungen durch Waffen im Zeitraum von 2010 bis 2018 dokumentiert und ausgewertet werden. Alle 7 Patienten waren männlich. Das Alter betrug im Mittel 44 ± 27,5 Jahre. Fünfmal kam es durch einen Suizidversuch zu den Verletzungen, zweimal durch einen Unfall. Bis auf eine Schussverletzung mit einer Armbrust wurden Feuerwaffen verwendet. Viermal kam es dabei zu einem Steckschuss, zweimal zu einem Durchschuss und einmal zu einem Prellschuss. Ein Streifschuss wurde nicht beobachtet. Das Ergebnis der Sehschärfe für die betroffenen Augen lag einmal bei einer einseitigen und einmal bei einer beidseitigen Amaurose, bei einem Patienten bei 1/35 MTV und bei 4 Patienten zwischen 0,2 und 0,7. Kein Patient ist an den unmittelbaren Folgen der Schussverletzung gestorben. Die Tab. 1 fasst die Ergebnisse zusammen. Im Folgenden sind die Krankengeschichten der 7 Patienten dargestellt.

**Table 1:** 

Fallnummer	1	2	3	4	5	6	7
Vorstellung	07/2010	06/2014	07/2014	01/2016	04/2016	05/2018	06/2018
Geschlecht	m	m	m	m	m	m	m
Alter	77 Jahre	18 Jahre	32 Jahre	72 Jahre	18 Jahre	22 Jahre	70 Jahre
Ursache	Suizidversuch	Suizidversuch	Unfall (Fremdverschulden)	Suizidversuch	Unfall (Fremdverschulden)	Suizidversuch	Suizidversuch vor 2 Tagen
Auslöser	Metastasiertes Prostatakarzinom, Ehefrau kurz zuvor verstorben	Zustand nach verursachtem Verkehrsunfall unter Alkoholeinfluss	„Beim Spielen passiert“	Metastasiertes Prostatakarzinom	„Von Freund versehentlich angeschossen worden“	Schwere depressive Episode	Schwere depressive Episode
Waffe (Projektil)	Faustfeuerwaffe (Pistole, 7 mm Kaliber)	Luftgewehr	Luftpistole	Langwaffe (Gewehr)	Luftgewehr	Armbrust (Bolzen)	Faustfeuerwaffe (Pistole, 9 mm Kaliber)
Schussart	Durchschuss	Steckschuss	Steckschuss	Durchschuss	Steckschuss	Prellschuss	Steckschuss
Schusskanal	Eintritt: Schläfe links – durch Orbita – oberhalb des Bulbus – unterhalb des Orbitadachs – weiter unter Haut – Austritt: Glabella	Projektil im linken Orbitatrichter	Eintritt: links frontal-orbital am Limbus nasal inferior mit parabulbärer Projektillage, keine neurokraniale Beteiligung	Eintritt linke Schläfe – horizontaler Durchtritt durch beide Orbitae – Austritt rechte Schläfe	Eintritt lateroorbital rechts – Projektil steckt im temporalen Oberlid	Aufschlagstelle: frontal parabulbär auf mediale Orbita	Eintritt: Gaumen – über rechte Kieferhöhle – über lateralen Orbitapfeiler – in die frontotemporale Kalotte – Projektil subgaleal
Bestkorrigierter Visus bei Vorstellung	RA: 0,4 LA: 0,2	Nicht zu erheben (intubiert)	RA: 1,0 LA: 0,05	Nicht zu erheben (intubiert)	RA: 0,7 LA: 0,7	RA:0,63 LA: 0,16	RA: HBW LA: 0,6
Verletzungsmuster	Weichteiltrauma mit Orbita- und Monokelhämatom, Vorderkammerblutung, Pupillenentrundung, kein Hinweis auf Bulbusruptur oder knöcherne Verletzung	Monokelhämatom Exophthalmus Retrobulbärhämatom Traumatische Mydriasis Stauungspapille mit Blutungen Verdacht auf Aderhautruptur	Schwere perforierende Bulbusverletzung mit Ablatio, Iris- und Linsenverlust ohne Hornhautbeteiligung	R: Bulbusruptur, fehlende intraokulare Gewebe, Verdacht auf Ruptur des N. opticus L: Avulsio nervi optici, Trümmerfraktur des Mittelgesichts, komplexe Frakturen beider Orbitae	Weichteilschwellung, Contusio bulbi, Glaskörperblutung, temporaler Netzhautriss, begleitende Amotio retinae, Prellmarke der Sklera, Verletzung a.e. indirekt durch Stoßwelle bedingt	Netzhautruptur, schwerste Contusio bulbi	Traumatische Mydriasis Verdacht auf traumatische Optikusneuropathie, Kalottenfrakturen, Orbitafrakturen, offene Gaumenwunde
Maßnahmen	Keine ophthalmologischen Maßnahmen	Systemische Antibiose und Steroide, lokale augendrucksenkende Therapie; Entlastung des Retrobulbärhämatoms durch MKG; Entfernung des Projektils durch Neurochirurgie	Primäre operative Wundversorgung mit Projektilentfernung Kryopexie, Lensektomie und Silikonöltamponade	R: Skleranähte + Bulbustonisierung mit Viskoelastikum L: Rücklagerung des Restbulbus und Tarsorrhaphie Bds. infauste okuläre Prognose	Wundexploration in ITN, Cerclage + Kryo + Bindehaut- und Hautnaht Projektil nicht auffindbar, sekundäre Projektilentfernung 2 Tage später	Operative Erstversorgung mit Sklerainspektion. Ausschluss Bulbuseröffnung und NH-Kryo	Systemische Steroide und Antibiose; Entfernung des Projektils durch Neurochirurgen; Extraktion zerstörter Zähne und Mund-Antrum-Deckung durch MKG
Verlauf	LA: 0,2 bei Entlassung Patient überlebte	LA: Amaurose, Hebungsdefizit Patient überlebte	LA 0,6 bei Zustand nach sek. Artificial-Iris und Sekundärlinsenimplantation Patient überlebte	R/L: im Verlauf bds. Enukleation Patient überlebte	RA: 0,2 bei Entlassung Patient überlebte	LA: 0,7, gute Rehabilitation Patient überlebte	RA: 1/35 MTV, Exotropie, Netzhautatrophie, Anisokorie Patient überlebte
Abbildung	Abb. 1	Abb. 2a	–	Abb. 3	Abb. 2b–d	Abb. 4	Abb. 2e–g

### Fall 1 (Abb. 1)

Ein 77-jähriger Patient in reduziertem Allgemeinzustand wurde nach einem Suizidversuch durch eine gegen sich gerichtete Faustfeuerwaffe vorgestellt. Er war wach, orientiert und ansprechbar und konnte detailliert Auskunft über das Geschehen geben: Der Patient litt an einem weit fortgeschrittenen Prostatakarzinom mit multiplen Knochenmetastasen in einem palliativen Behandlungsregime. Zudem war vor Kurzem seine Frau verstorben. Die Pistole mit einem Kaliber von 7 mm wurde nach Angaben des Patienten an der linken Schläfe mit ca. 10 cm Abstand und Zielrichtung Glabella ausgelöst. Unmittelbar nach dem Schuss wollte er die Pistole nochmals auslösen, brachte jedoch die Kraft nicht mehr auf.

**Figure 1:**
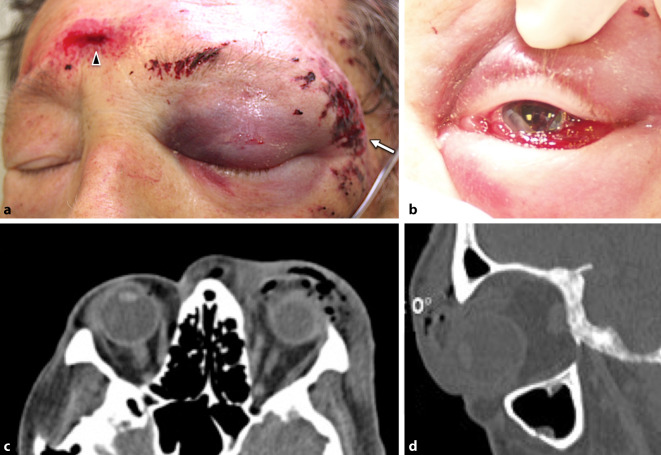

Das Projektil durchschlug an der Eintrittspforte die Schläfenhaut mit Schmaucheinsprengungen. Die Austrittsöffnung war an der mittigen Stirn zu erkennen.

Die klinische Untersuchung zeigte am linken Auge einen Visus von 0,2, der intraokulare Augendruck lag bei 23 mm Hg. Es fand sich ein ausgeprägtes Monokelhämatom mit Lidschwellung, Sanguiszellen in der vorderen Augenkammer, die Pupille war leicht entrundet und die leicht getrübte Linse befand sich in normaler Position. Fundoskopisch war die Papille vital und randscharf und die periphere Netzhaut unauffällig. Anzeichen für eine Bulbusperforation oder Ruptur fanden sich nicht.

In der kranialen Computertomographie (cCT) führte der Schusskanal des Projektils durch die Orbita oberhalb des Bulbus unterhalb des Orbitadaches und trat unterhalb am medialen Arcus supraorbitalis unter die Haut und bildete an der Glabella die Austrittspforte. Knöcherne Strukturen wurden nicht verletzt, der Bulbus, der Sehnerv und andere wichtige Strukturen verfehlt. Aufgrund des Weichteiltraumas kam es zu einem ausgedehnten Lid- und Orbitahämatom mit multiplen Lufteinschlüssen.

### Fall 2 (Abb. 2a)

Bei einem 18-jährigen männlichen Patient ist es unter Alkoholeinfluss zu einem Verkehrsunfall sowie einer anschließenden selbst zugefügten Schussverletzung mit einem Luftgewehr im Bereich des Schädels links gekommen. Es lag kein Anhalt für Fremdverschulden vor.

**Figure 2:**
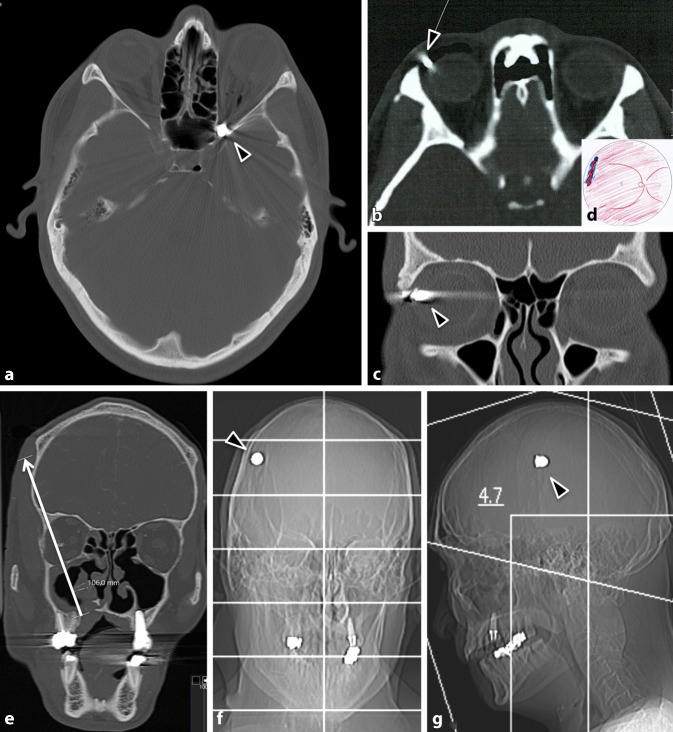

Initial war eine Visuserhebung bei dem intubierten, beatmeten Patienten nicht möglich. Der Augeninnendruck war palpatorisch erhöht. Klinisch zeigte sich am linken Auge ein Monokelhämatom, ein Exophthalmus mit injizierter, chemotischer Bindehaut, die Pupille in traumatischer Mydriasis ohne Lichtspiel bei ansonsten unauffälligen vorderen Augenabschnitten. Fundoskopisch fielen eine gestaute Papille mit Blutungen sowie gestaute Gefäße auf.

In der cCT ließen sich das Projektil im linken Orbitatrichter sowie ein Retrobulbärhämatom links mit Protrusio bulbi darstellen.

Es erfolgte eine umgehende Entlastung des Retrobulbärhämatoms durch die Kollegen der MKG. Dabei gelang es nicht, das Projektil aus der Orbitaspitze zu entfernen.

Nach Extubation erfolgte am Folgetag eine Re-Evaluation: Der Visus am linken Auge lag bei nulla lux, der Augeninnendruck betrug 31 mm Hg. Weder die direkte noch die indirekte Lichtreaktion war intakt, die Bulbusmotilität war vollständig aufgehoben. Fundoskopisch zeigten sich eine Glaskörperblutung inferior, ein Netzhautödem und der Verdacht auf eine Aderhautruptur bis an den unteren Gefäßbogen reichend mit freiliegender Sklera bei vermutlich druckwellenbedingter Ursache. Es bestand kein Anhalt für eine Bulbusperforation.

Das Projektil konnte durch die Neurochirurgen mittels pterionaler Trepanation, anteriorer Clinoidektomie, Entfernung des Orbitadaches und Hinterwandorbitotomie 2 Tage später entfernt werden.

Postoperativ persistierte die Amaurosis links ohne weitere neurologische Defizite, und die Bulbusmotilität erholte sich bis auf ein Hebungsdefizit.

### Fall 3

Ein 32-jähriger Mann stellte sich wegen einer unfallbedingten fremdverschuldeten Schussverletzung mit einer Luftpistole in unserer Klinik vor. Die Eintrittspforte lag in der medialen inferioren linken Orbita und führte dabei zu einer perforierenden Bulbusverletzung am Limbus mit Iristeilverlust über eine Uhrzeit. Primär wurden eine Wundversorgung mit Projektilentfernung aus der anterioren Orbita, Kryopexie, Lent- und Vitrektomie mit Silikonöltamponade durchgeführt. Postoperativ bestanden partielle Irisdefekte, Aphakie sowie Netzhaut- und Aderhautnarben. Nach sekundärer Versorgung mit einer sklerafixierten Intraokularlinse und einer sklerafixierten künstlichen Iris (Artificial-Iris, HumanOptics, Erlangen, Deutschland) stieg der Visus zuletzt auf 0,6 an.

### Fall 4 (Abb. 3)

In suizidaler Absicht hatte sich ein 72-jähriger Patient mit einem Gewehr in die linke Schläfe geschossen. Er war bei Vorstellung intubiert und beatmet. An der Schläfe links war die Eintrittspforte des Projektils zu erkennen, die Austrittspforte befand sich kontralateral an der rechten Schläfe. CT-morphologisch zeigten sich eine Trümmerfraktur des Mittelgesichts mit komplexer Fraktur beider Orbitae, der Ethmoidalzellen, des Sinus maxillaris und des Arcus zygomaticus rechts sowie eine Nasenbeinfraktur. Es bestanden eine Parenchymblutung rechts temporal sowie eine Schädelbasisfraktur im Sinne eines offenen Schädel-Hirn-Traumas. Des Weiteren bestand der Verdacht auf eine Ruptur des N. opticus rechts. Die Bulbuskontur war beidseits deformiert mit inhomogener Binnenstruktur und multiplen Fremdkörpern.

**Figure 3:**
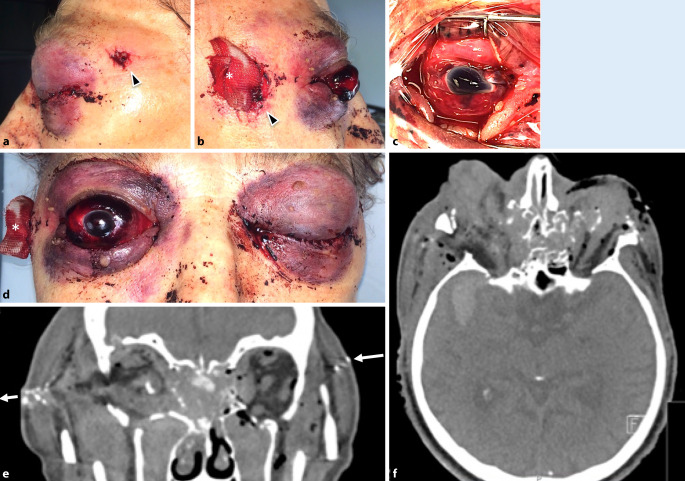

Klinisch zeigten sich am rechten Auge eine massive Lidschwellung, ein Hyposphagma sowie eine vollständig blutgefüllte Vorderkammer. Von 9 Uhr über 12 Uhr bis 4 Uhr war am Limbus eine Rupturlinie erkennbar, sodass die Hornhaut fast vollständig aufgeklappt werden konnte. Die Rupturlinie setzte sich bei 3 Uhr und 12 Uhr nach posterior fort. Die Sklera war exponiert, und intraokulares Gewebe fehlte. Die Sklera wurde mit multiplen Nähten adaptiert und der Bulbus verschlossen. Anschließend erfolgte die Tonisierung mit Viskoelastikum. Aufgrund der massiven Verletzungen wurde auf eine primäre Vitrektomie verzichtet. Bei fehlendem intraokularem Gewebe (Glaskörper, Netzhaut, Ziliarkörper, Linse) bestand eine infauste Prognose.

Am linken Auge imponierte eine massive Protrusio bulbi, sodass die Lider hinter dem Bulbus zum Liegen kamen. Der Bulbus war weich und mit Sanguis gefüllt. Eine Testung der Lichtreaktion war nicht möglich. Bei radiologischem und ophthalmologischem Verdacht auf eine Avulsio n. optici erfolgte der Entschluss gegen eine umfangreiche chirurgische Primärversorgung. Zum Schutz der Hornhaut wurden lediglich bis zur Abschwellung des umgebenden Gewebes eine Rücklagerung des Restbulbus und Tarsorrhaphie durchgeführt. Im Verlauf war eine beidseitige Enukleation unvermeidbar.

### Fall 5 (Abb. 2b–d)

Die erste konsiliarische Vorstellung des 18-jährigen Patienten erfolgte durch die Kollegen MKG, wo sich der Patient initial notfallmäßig mit Fremdkörpereinsprengung periorbital rechts vorstellte. Anamnestisch gab der Patient an, er sei versehentlich von einem Freund mit einem Luftgewehr angeschossen worden.

Klinisch zeigte sich eine ca. 0,5 cm messende Eintrittswunde lateroorbital rechts mit einer dezenten Weichteilschwellung. Des Weiteren stellte sich eine schwere Contusio bulbi mit Glaskörperblutung und temporalem Netzhautriss mit begleitender Netzhautablösung dar. Der Visus lag beidseits bei 0,7 sc. Der intraokulare Druck betrug 18 mm Hg.

In Vollnarkose erfolgte eine Wundexploration, bei der sich nach Eröffnung der Bindehaut und Anschlingen der 4 geraden Augenmuskeln eine intakte Sklera mit Prellmarke bei 9:30 Uhr mit episkleralem Blutungsring darstellte. Ein Geschoss war weder sicht- noch tastbar. Auch mittels Magneten war es nicht extrahierbar. Da die Sonde bei der Sondierung des Schusskanals nicht bis zum Bulbus reichte lag der Verdacht nahe, dass lediglich die Stoßwelle des Geschosses den Bulbus erreichte. Ophthalmoskopisch zeigte sich ein oranaher Riss von 7–9 Uhr mit eingerissener Netzhaut, welcher nach inferior in eine präretinale und Glaskörperblutung überging. Eine Cerclage mit Kryopexie wurde durchgeführt.

Postoperativ lag die Netzhaut bei Glaskörpernachblutung sonographisch an. Weiterhin zeigte sich das Projektil mit einem Durchmesser von ca. 3 mm im temporal oberen Lidquadranten. Mittels cCT konnte die Position bestätigt werden. In einem Sekundäreingriff erfolgte die endgültige Entfernung des Fremdkörpers.

### Fall 6 (Abb. 4)

Der 22-jährige männliche Patient wurde in unserer Notfallambulanz in Begleitung der Polizei vorgestellt, nachdem sein linkes Auge mit einem Pfeil einer Armbrust in suizidaler Absicht getroffen wurde. Sowohl die Armbrust als auch das Projektil wurden vorgezeigt.

**Figure 4:**
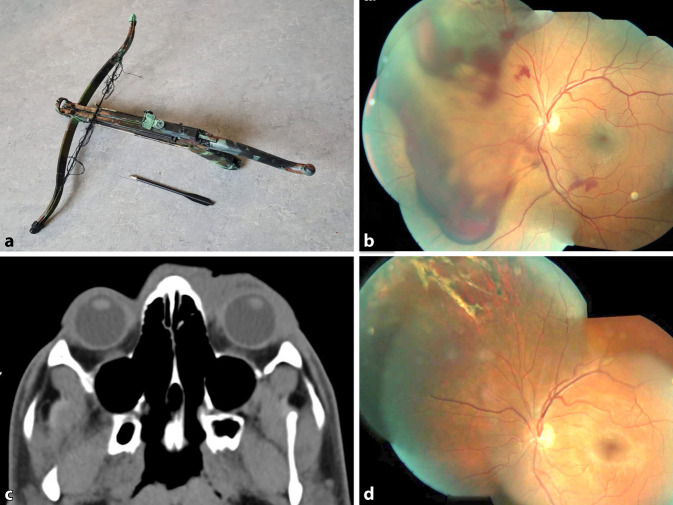

Der Visus betrug 0,16 cc am betroffenen Auge, der Augendruck lag bei 17 mm Hg. Morphologisch zeigte sich ein Lidhämatom bei sonst unauffälligem vorderem Augenabschnitt. Fundoskopisch zeigte sich ein Netzhautriss bei 11 Uhr mit Blutung und Berlin-Ödem. Daraufhin erfolgte eine Narkoseuntersuchung mit Netzhautkryokoagulation des Netzhautrisses. Die intraoperative Inspektion des Bulbus ergab keinen Hinweis auf eine Bulbusperforation, weitere Blutungen oder Dehiszenzen. Postoperativ erholte sich der Visus und betrug 0,7 cc am betroffenen Auge.

### Fall 7 (Abb. 2e–g)

Die Einlieferung des 70-jährigen Patienten in die Klinik erfolgte, nachdem er sich in suizidaler Absicht 2 Tage zuvor mit einer Pistole (Kaliber 9 mm) mit dem Lauf im Mund in den Kopf geschossen hatte und von Bekannten aufgefunden worden war.

Bei Aufnahme zeigte sich der Patient wach und orientiert. Der Handtafelvisus lag rechts bei vorbekannter Katarakt bei HBW, am linken Auge bei 0,6 mit Korrektur. Die Pupille rechts zeigte sich mittelweit, nicht lichtreagibel, die linke Pupille war direkt und indirekt lichtreagibel. Soweit nach Hornhautreflexen beurteilbar, bestand Orthophorie, die Motilität war grob orientierend frei. Das Projektil ließ sich rechts temporal subkutan tasten. Die Eintrittspforte am rechten Gaumen war eitrig belegt.

Fundoskopisch war der Einblick kataraktbedingt reduziert, die Papillen schienen randscharf und vital, die Makula und Netzhaut waren – soweit einsehbar – grob unauffällig.

In der Zusammenschau der Befunde wurden die Diagnose einer traumatischen Mydriasis und der Verdacht auf eine traumatische Optikusneuropathie (TON) rechts gestellt.

Der Schusskanal erstreckte sich klinisch von der Mundhöhle nach rechts kranial über den harten Gaumen, die rechte Kieferhöhle und den lateralen Orbitapfeiler bis in die frontotemporale Kalotte. In der nativen cCT zeigte sich eine Parenchymblutung rechts frontal sowie rechts temporal mit deutlichem perifokalem Ödem, Pelottierung und deutlicher Einengung des rechten Seitenventrikels. Das Projektil ließ sich extrakraniell subgaleal rechts frontal lokalisieren. Des Weiteren stellten sich eine mehrfragmentäre Kalottenfraktur rechts frontal und temporal und eine Fraktur der lateralen Orbitawand dar. Der rechte Orbitaboden sowie der rechte Orbitapfeiler zeigten sich insgesamt destruiert mit Dislokation multipler kleinster knöcherner Fragmente nach intraorbital. Diese Knochensplitter mit bis zu 6 mm Länge lagen retrobulbär-intrakonal und am Unterrand des M. rectus lateralis. Zudem ergab sich der Verdacht auf winzige Knochensplitter am Unterrand des N. opticus, kurz nach Austritt aus dem Bulbus.

Im Rahmen der Erstversorgung mit neurochirurgischer Intensivbetreuung erfolgte die Bergung des Projektils rechts frontal mit Entfernung der Knochenfragmente, Duraversorgung und Pallacos-Plastik. Der Patient stand im Verlauf einer psychiatrischen Therapie offen gegenüber. Daher erfolgte die Verlegung in die Psychiatrie mit augenärztlicher und kieferchirurgischer Anbindung. Bei der kieferchirurgischen Versorgung erfolgte eine Extraktion der zerstörten Zähne 15 und 16 sowie Mund-Antrum-Verbindung-Deckung mit palatinalem Rotationslappen und Bichat- und Rehrmann-Transplantat.

Unter Fortführung der systemischen Antibiose erfolgten von ophthalmologischer Seite zunächst weitere Kontrollen. Im Verlauf betrug der Visus am rechten Auge cc 1/35 MTV, am linken Auge 0,6 cc. Der Augendruck betrug 9 mm Hg beidseits.

OCT-morphologisch stellte sich am rechten Auge eine Netzhautatrophie dar.

## Diskussion

Insgesamt sind Schussverletzungen hierzulande und insbesondere im Bereich der Augenheilkunde sehr selten. Für Schussverletzungen besteht ebenso wie für Stichverletzungen keine Meldepflicht. Außerdem ist der Arzt nach § 9 der Berufsordnung zur Schweigepflicht gehalten. Der Arzt ist zur Offenbarung nur dann befugt, wenn er von der Schweigepflicht entbunden wurde oder soweit die Offenbarung zum Schutze eines höherwertigen Rechtsgutes erforderlich ist.

Eine wissenschaftliche Analyse gestaltet sich auch aufgrund des sehr heterogenen Verletzungsmuster, der meist schwierigen Anamnese und der Involvierung anderer Fachdisziplinen als schwierig. In vielen Fällen kommt es aufgrund einer zerebralen Beteiligung zum letalen Ausgang [5, 17]. Der Kopf wird für suizidale Schussverletzungen mit 82 % als stark bevorzugtes Ziel ausgewählt [9]. Nachvollziehbarerweise wurden nur diejenigen Patienten in der Augenklinik vorstellig, die ihre Verletzungen bis zur Konsultation überlebten. Dabei wurde die Schusswaffe meist an der Schläfe angesetzt und das Projektil abgegeben. Häufig werden für Suizidversuche Pistolen mit einem Kaliber von 5–9 mm eingesetzt. Die Energie und Geschwindigkeit (300–500 m/s) dieser Geschosse reicht in der Regel für eine Durchdringung der Schädelkalotte aus [18].

Kein Patient in unserem Kollektiv wurde vorstellig, bei dem der Schusskanal das Neurokranium betraf; alle Patienten zeigten die Verletzungen nur im Viszerokranium. Die Schwere der Verletzung und die betroffenen Strukturen waren maßgeblich von dem Weg des Projektils abhängig [12]. Schäden werden hierbei entweder direkt durch die Projektile selbst, indirekt durch die Energieübertragung des Projektils oder die Druckwelle auf das Umgebungsgewebe verursacht [6, 10]. Dabei bestimmen die Art der Waffe, das Kaliber des Projektils sowie dessen Form, dessen Eintrittswinkel und Geschossverlauf entscheidend das Ausmaß der Verletzung.

Die Verletzungsmuster bei Schussverletzungen erklären sich wie folgt:

Beim Durchdringen des Körpers zerdrückt, zerschneidet und zerstört das Projektil das Gewebe und hinterlässt einen Schusskanal [3]. Dieser Schusskanal erweitert sich durch ein Taumeln des Projektils. Zusätzlich vergrößert sich der Schusskanal durch Kavitation und eine ballistische Druckwelle aufgrund der kinetischen Energie des Projektils weiter. Es kommt zu einer radialen Beschleunigung des Gewebes mit Wundkanalexpansion und zu einer kurz bestehenden temporären Wundhöhle [2, 11, 21].

Bei nicht deformierenden Projektilen aus Handfeuerwaffen ist dieser Effekt deutlich geringer als bei nicht deformierenden Projektilen aus Gewehren. Der Grund liegt in der geringeren Energie der Projektile durch eine geringere Geschwindigkeit und Masse. Zum anderen liegt dies aber auch an der geringeren Länge der Projektile, sodass beim Gieren (= Drehbewegung um die vertikale Achse) der Projektile eine geringere Kontaktfläche zum getroffenen Gewebe besteht [8]. Wird durch die Entstehung der temporären Wundhöhle das betroffene Gewebe über seine Elastizität hinaus gedehnt, zerreißt es [3, 21]. Bei Verletzungen des Bulbus kommt neben der direkten mechanischen Affektion durch das Projektil selbst der Effekt der hydrodynamischen Sprengwirkung dazu. Hierunter versteht man den Effekt, der dazu führt, dass die Energie des Geschosses beim Auftreffen auf flüssigkeitsgefüllte Hohlorgane zum Aufplatzen der Organwandung führt. Daraus ergibt sich, dass flüssigkeitsgefüllte Organe im Vergleich zu luftgefüllten Organen mit einem deutlich größeren Schaden reagieren [15, 21, 23]. Dieses Phänomen ist insbesondere in unserem vierten Fall nachzuvollziehen und ist auch bereits bei Medicke et al. beschrieben worden [16]. In diesen Fällen ist es besonders schwierig, rekonstruktive Maßnahmen durchzuführen. Oftmals kann nur eine vorübergehende Wundversorgung bei infauster Prognose angestrebt werden, bis sekundär eine Enukleation erfolgen muss [12]. Eine primäre Enukleation sollte aber selbst bei aussichtslosen Befunden möglichst vermieden werden, da die Genesung oftmals schwer vorherzusagen ist. Eine operative Versorgung auch schwerverletzter Augen kann sich immer noch lohnen [13]. Außerdem gibt es Patienten das bleibende Gefühl, wenigstens alles operativ Mögliche versucht zu haben. Prinzipiell hängen bei schweren Augenverletzungen der anatomische und funktionelle Erfolg entscheidend vom Zeitpunkt der Erstversorgung als auch von der sekundären Rekonstruktion ab [22]. In Anbetracht der heterogenen Verletzungsmuster ist der ideale Zeitpunkt für eine sekundäre Rekonstruktion in unserem Kollektiv sehr individuell zu treffen. Bei einer Schussverletzung können verschiedene Strukturen des Auges geschädigt werden. Schwerwiegende Schäden können auch dann auftreten, wenn ein Hochgeschwindigkeitsprojektil auf den Bulbus auftrifft, diesen aber nicht durchdringt. Oftmals kommt es hierbei zu einer Ruptur der Aderhaut und der darüber liegenden neurosensorischen Netzhaut, was dann als Retinopathia sclopetaria beschrieben wird (s. Fall 2, 5 und 7) [14].

Alle Schusswunden müssen prinzipiell als kontaminiert angesehen werden. Postakzidentielle Infektionen wurden – vermutlich durch die Gabe einer prophylaktischen intravenösen Breitspektrumantibiose nach Maßgabe aller beteiligten Fachdisziplinen – in unserer Serie in keinem Fall beobachtet.

Die Mehrheit (5 von 7) der orbitalen und okulären Schussverletzungen aus unserer Kohorte rührte von suizidaler Absicht her. Zudem waren alle Patienten männlich. Diese Geschlechterverteilung bei Schussverletzungen spiegelt sich auch in der Literatur wider [13, 23]. Auffallend war das Alter der Patienten mit 2 Häufigkeitsgipfeln: Ein junges Kollektiv von 18 bis 32 Jahren (*n* = 4, 2 Unfälle und 2 Suizidversuche) und ein älteres mit 70 bis 77 Jahre (*n* = 3, alle Suizidversuche). Bei den älteren Patienten spielten im Rahmen einer depressiven Episode schwere Belastungsfaktoren wie maligne Grunderkrankungen im fortgeschrittenen Stadium oder der Verlust eines nahen Angehörigen als Auslöser für den Suizidversuch eine Rolle. Bei den jüngeren Patienten rührte die Schussverletzung entweder durch einen Unfall oder Suizidversuch her. Auch bei den jungen Patienten mit Suizidversuch waren psychosoziale Belastungsfaktoren vorhanden.

## Schlussfolgerung

Schussverletzungen sind in Deutschland selten und haben meist einen suizidalen Hintergrund, wobei hinter jedem Ereignis immer ein individuelles Schicksal steht. Eine überlebte Schussverletzung hat je nach Schusskanal schwerwiegende Folgen für die intraorbital gelegenen Strukturen. Die meisten Verletzungen bedürfen einer interdisziplinären Zusammenarbeit, wie insbesondere unser Fall 7 zeigt. Ein Rekonstruktionsversuch lohnt sich stets.

## Fazit für die Praxis

- Orbitale Schussverletzungen sind selten und werden meistens in suizidaler Absicht herbeigeführt.
- Eine interdisziplinäre Kooperation von Ophthalmo‑, Neuro‑, MKG- bzw. HNO-Chirurgen und Psychiatern ist notwendig.
- Überlebte Schussverletzungen im Gesichtsbereich mit okulärer Beteiligung führen oft zu schwersten bleibenden Sehbehinderungen.
- Die Auswahl und das Ausrichten der Waffe und damit die Planung zur Bildung des Schusskanals sowie die freigesetzte Energie sind maßgeblich für die Verletzungsschwere verantwortlich.
